# Biointerfacial self-assembly generates lipid membrane coated bacteria for enhanced oral delivery and treatment

**DOI:** 10.1038/s41467-019-13727-9

**Published:** 2019-12-19

**Authors:** Zhenping Cao, Xinyue Wang, Yan Pang, Shanshan Cheng, Jinyao Liu

**Affiliations:** 1 Institute of Molecular Medicine, State Key Laboratory of Oncogenes and Related Genes, Shanghai Cancer Institute, Renji Hospital, School of Medicine, Shanghai Jiao Tong University, Shanghai, 200127 China; 2 Department of Ophthalmology, Shanghai Ninth People’s Hospital, School of Medicine, Shanghai Jiao Tong University, Shanghai, 200011 China

**Keywords:** Drug delivery, Cell delivery, Biomaterials

## Abstract

The gut microbiota represents a huge community of microorganisms that play essential roles in immune modulation and homeostasis maintenance. Microbiota transplantation is an important approach to prevent and treat disease as it can inhibit pathogen colonization and positively modulate bacterial composition. However, the development of oral bacterial therapeutics has been restricted by low bioavailability and limited retention in the gastrointestinal tract. Here, we report a simple yet highly efficient method to coat gut microbes via biointerfacial supramolecular self-assembly. Coating can be performed within 15 min by simply vortexing with biocompatible lipids. Bacteria coated with an extra self-assembled lipid membrane exhibit significantly improved survival against environmental assaults and almost unchanged viability and bioactivity. We demonstrate their enhanced efficacies in oral delivery and treatment using two murine models of colitis. We suggest that biointerfacial supramolecular self-assembly may provide a unique platform to generate advanced bacterial therapeutics for the treatment of various diseases.

## Abstract (web summary)

Oral microbiota delivery is an approach to treat and prevent disease but suffers from low retention and bioavailability. Here the authors report on a lipid coating to protect against environmental assault maintaining viability and bioactivity of the bacteria and demonstrate effective application in a colitis model.

## Introduction

The gut microbiota represents a huge community of microorganisms including archaea, bacteria, fungi, viruses and microeukaryotes that occupy the gastrointestinal (GI) tract^1,2^. Intensive research on the gut microbial community has provided growing evidence that some of the microorganisms play essential roles in immune modulation, the maintenance of homeostasis, and host health^3^. Disorders of the intestinal microbiota are relevant to a variety of diseases, such as diabetes, obesity, hypertensive heart disease, inflammatory bowel disease, and some cancers^4–7^. The delivery of probiotic bacteria to the microbiome is a promising way to prevent and treat disease. The introduced organisms can accumulate at intestinal site, inhibiting pathogen colonization and positively modulating the balance of bacterial composition, thus exerting beneficial effects^8,9^. Various forms of liquids, pills, enteric coatings, polymer gels, and dry powder have been developed for oral delivery of probiotic bacteria^10–12^. While these formulations have been successful in improving delivery efficiency, the use of gut microbes as oral therapeutics has been largely restricted by their limited treatment efficacy caused by low bioavailability and insufficient retention in the GI tract^11,13^.

The development of engineered gut microbes including genetically and chemically modified bacteria is an alternative to address some of these limitations^13,14^. For example, genetically engineered bacteria with increased stress tolerance have been produced to mediate the delivery of vaccines, drugs, and immunomodulators, or to directly kill specific pathogens or inhibit their virulence^15–17^. Chemical based surface modification and encapsulation using synthetic materials has been explored to improve viability and stability by preventing probiotic bacteria from acid and enzyme insults in the GI tract^11,13,18,19^. However, despite their promise for oral delivery, few of these approaches have proven effective as prevention or treatment in humans or animals because they inevitably suffer from: inefficient survival in complex GI environments, such as variability in individuals’ gut microbiota, diet, drugs (e.g., antibiotics), strongly acidic gastric fluid, digestive enzymes, and bile salts, which cause massive death of the bacteria; limited colonization and proliferation in the GI tract as the coatings prevent direct contact of bacteria with the intestinal mucosa, resulting in short retention and insufficient level of the bacteria to function adequately; and multiple preparation steps and tedious separation procedures, which render them unfriendly for manufacturing scale-up. New strategies are hence needed to address these limitations for the development of oral bacterial therapeutics.

Here, we report a simple yet highly efficient method to generate super gut microbes, a set of lipid membrane coated bacteria (LCB) which can address all these limitations, by wrapping bacteria with an extra lipid membrane via biointerfacial supramolecular self-assembly. We demonstrate the rapid preparation of LCB in less than 15 min by simply vortexing bacteria with biocompatible lipids. The resultant LCB exhibit: significantly improved survival against various extreme conditions including strong acids and alkalis, simulated gastric and intestinal fluids, antibiotics, and ethanol, which is attributed to the presence of the self-assembled lipid membrane; almost unchanged viability and bioactivity as the coating lipid membrane can disassemble after targeting disease sites, which leads to unaffected mucosal adhesion, colonization, and proliferation; simple yet highly efficient preparation and the use of Food and Drug Administration (FDA) approved materials that can facilitate their manufacturing scale-up and subsequent translation. We further show that LCB have almost three-times higher survival in the mouse stomach and more than four-times higher bioavailability in the gut compared with uncoated bacteria. The enhanced reservation in the gut maintains for up to 4 days post-administration. We also demonstrate that LCB achieve significantly increased efficacies both in prevention and treatment in *Salmonella typhimurium* (STm) and dextran sulfate sodium (DSS) induced colitis mouse models. We propose that super gut microbes coated with self-assembled lipid membranes could provide a unique platform for advanced delivery of bacteria for a myriad of biomedical applications.

## Results and discussion

### Preparation and characterization of LCB

**Fig. 1:**
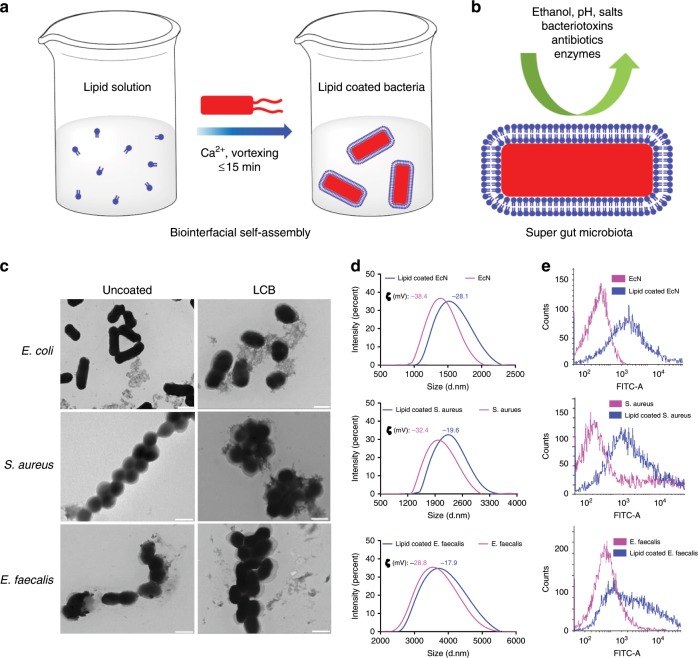
Preparation and characterization of LCB. **a** Schematic illustration of the preparation of lipid membrane coated bacteria by biointerfacial supramolecular self-assembly. **b** The presence of coating membranes endows probiotic bacteria with exceptional resistance to various harsh environmental conditions. **c** Representative TEM images of uncoated bacteria and LCB. Scale bar: 1 μm. **d** Size distribution and Zeta potential of uncoated bacteria and LCB measured by DLS. Data are presented as the means ± SD (*n* = 3). e Flow cytometric analysis of FITC-labeled LCB. Unlabeled LCB were used as controls.

As a proof-of-concept study, we chose *Escherichia coli* Nissle 1917 (EcN), a well-known probiotic bacterium^20^, which has been widely used for treating a range of GI disorders and metabolic diseases^15,21–24^. EcN were coated by vortexing with dioleoylphosphatydic acid (DOPA) and cholesterol in calcium phosphate buffer (Fig. 1a). The use of calcium ions for the preparation of LCB is critical because they assist the self-assembly of DOPA on the negative surface of the bacteria. Cholesterol was used to further stabilize the self-assembled lipid membranes. The optimized molar ratio of DOPA to cholesterol was set at 4:1 which could isolate the coated EcN most consistently (Fig. 1b and Supplementary Fig. 1a, b). Transmission electron microscopy (TEM) images show a clear extra outer shell on the coated bacteria, is in stark contrast with the sharp edge of uncoated EcN (Fig. 1c). The coating membranes were visualized to be thicker than a usual lipid bilayer, which might be caused by the significant dehydration of the coated bacteria before TEM observation^25^. Bacteria with different morphologies including spherical *Staphylococcus aureus* (*S. aureus*) and elliptical *Enterococcus faecalis* (*E. faecalis*) could be similarly coated, showing the broad applicability of this approach to coat diverse strains. Dynamic light scanning (DLS) measurements show an increase in both size and zeta potential after coating with lipid membranes (Fig. 1d and Supplementary Fig. 1c–e). EcN, *S. aureus*, and *E. faecalis* enlarged to 1.2, 1.3, and 1.1-fold their initial sizes, respectively. The zeta potential of EcN, *S. aureus*, and *E. faecalis* increased, respectively, from −38.4 ± 3.2, −32.4 ± 4.5, and −28.8 ± 1.6 mV to −28.1 ± 3.4, −19.6 ± 3.4, and −17.9 ± 0.7 mV, which were presented as the means ± SD of three independent experiments. Moreover, the coatings were labeled with fluorescein isothiocyanate (FITC) by co-assembly of DOPA with FITC-polyethylene glycol modified lipid (distearoylphosphatidylethanolamine, 10% of molar ratio). The labeled LCB were measured by flow cytometry, exhibiting a dramatic increase in fluorescent intensity over uncoated bacteria, demonstrating the presence of coating membranes (Fig. 1e). The coating membranes labeled with Nile Red were also observed by laser scanning confocal microscopy (LSCM) (Supplementary Fig. 1f–h). Taken together, these results demonstrate the generality, simplicity, and high efficiency of using biointerfacial supramolecular self-assembly to coat bacteria.

**Fig. 2:**
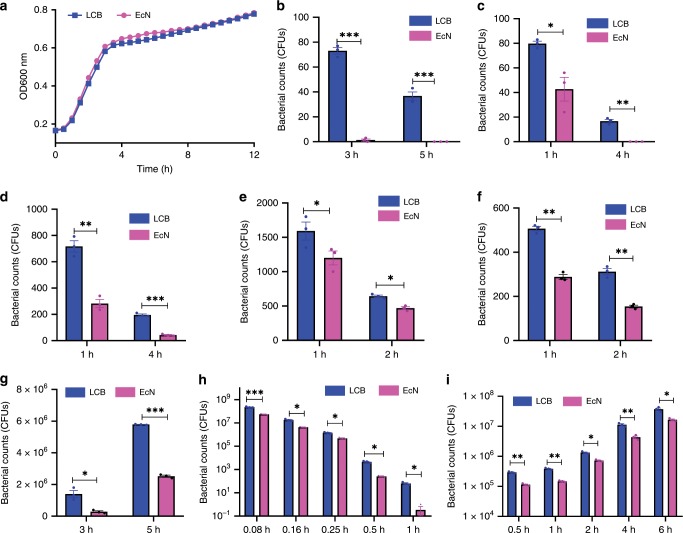
In vitro resistance of LCB against environmental assaults. **a** Growth curves of uncoated EcN and LCB cultured in LB medium at 37 °C and the OD_600_ was recorded at 30 min intervals using a microplate reader. **b**–**i** Equal amounts of uncoated EcN and LCB were exposed to the following: **b** an antibiotic cocktail of ampicillin and apramycin, **c** a strong acid (pH 2), **d** a strong alkali (pH 11), **e** 30% (v/v) ethanol, **f** 50% (v/v) ethanol, **g** 0.3 mg ml^−1^ bile salts, **h** SGF (pH 1.2) supplemented with pepsin and **i** SIF (pH 6.8) containing trypsin at 37 °C. After the indicated time points, 50 μl of each sample was washed twice with fresh LB, spread onto LB agar plates and incubated at 37 °C for 24 h before bacterial counting. Error bars represent standard deviation (*n* = 3). Significance was assessed using Student’s *t*-test, giving *p* values, *p < 0.05, **p < 0.01, ***p < 0.005.

To examine whether the coatings have effects on the viability and growth of EcN, bacterial viability was assessed using a cell counting kit-8 (CCK-8) assay. Interestingly, almost no difference was observed in viability between uncoated EcN and LCB either in Luria Bertani (LB) medium or LB with serum (Supplementary Fig. 2a and b). Moreover, LCB retained their ability to grow and divide when they were incubated in culture media. As shown in Fig. 2a and Supplementary Fig. 2c, LCB grew at a similar rate in LB and LB with serum media at 37 °C compared to uncoated EcN.

### In vitro resistance of LCB against environmental assaults

We then investigated the effect of coating membranes on the survival and growth of LCB in the presence of a number of anti-bacterial chemicals including antibiotics, strong acidity and alkalinity as well as ethanol. As shown in Fig. 2b, 50-times more coated bacteria survived after 3 h exposure to the antibiotic combination of ampicillin and apramycin compared with uncoated EcN. Following plating, counts of surviving bacteria fell by only 50% at incubation times extending from 3 to 5 h, while complete death was observed for uncoated EcN during this time. Similar results were obtained after exposing LCB to strong acidic (pH 2.0) and alkali (pH 11) conditions, where LCB showed greatly enhanced survival (Fig. 2c, d). Ethanol has a strong bactericidal activity and is able to kill bacteria directly by dehydrating and denaturing proteins^26,27^. In the presence of coating membranes, bacterial survival was at least twofold higher than that of uncoated EcN even when the concentration of ethanol was increased to 50% (v/v) (Fig. 2e, f). On the basis of these results, we speculated that LCB may also have enhanced survival in GI tract environments. Therefore, the survival of LCB was further evaluated in bile salts (0.3 mg ml^−1^), simulated gastric fluid (SGF) supplemented with pepsin (pH 1.2) and simulated intestinal fluid (SIF) containing trypsin (pH 6.8). As shown in Fig. 2g–i, LCB showed significantly improved tolerance to these synthetic GI tract environments compared to uncoated EcN. Additionally, *Bacillus subtillus* (BS), appeared similar enhanced survival after encapsulation (Supplementary Fig. 3), demonstrating the protection is translatable to other probiotic strains.

**Fig. 3:**
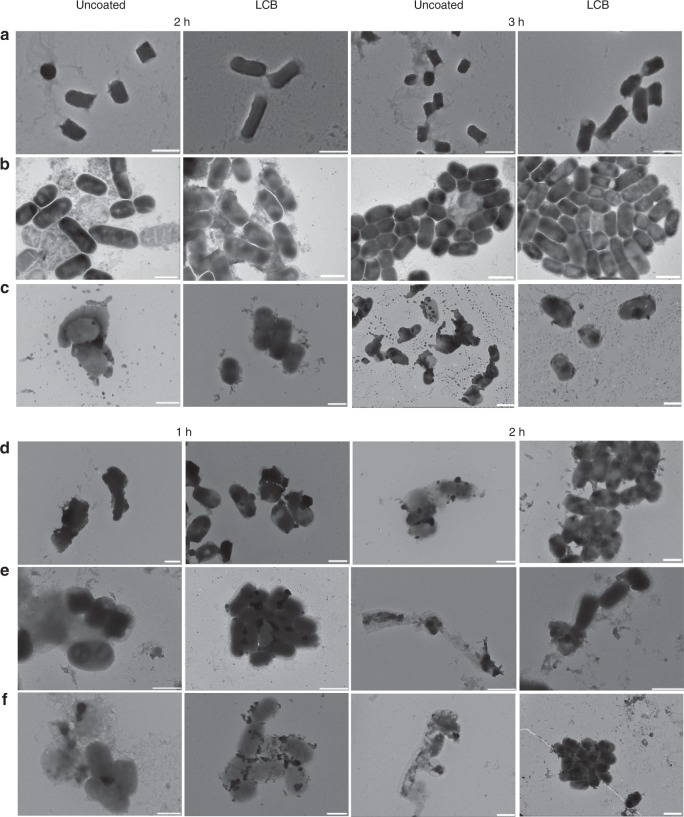
Morphology of LCB after exposure to environmental assaults. **a**–**f** Typical TEM images of uncoated EcN and LCB cultured in **a** SGF, **b** SIF, **c** antibiotics, **d** pH 11, **e** pH 2, and **f** 30% ethanol at 37 °C for 1, 2 and 3 h, respectively. Scale bar: 1 μm. A drop of bacterial solution was deposited onto a carbon-coated copper grid. The sample was further rinsed with double distilled H_2_O twice and subsequently dried completely in air before observation.

To confirm that the enhanced resistance observed was attributable to the presence of coating membranes, TEM was used to examine bacterial morphology after exposure to these harsh conditions. As shown in Fig. 3a, b, LCB remained intact following 3 h exposure to SGF and SIF, while uncoated EcN appeared aberrant, and were further damaged following extension of incubation time. The ability of the lipid coating to maintain the integrity of bacteria was also observed following exposure to bile salts, antibiotics, ethanol as well as strong acidity and alkalinity (Fig. 3c–g).

### In vivo resistance of LCB against GI tract environment

**Fig. 4:**
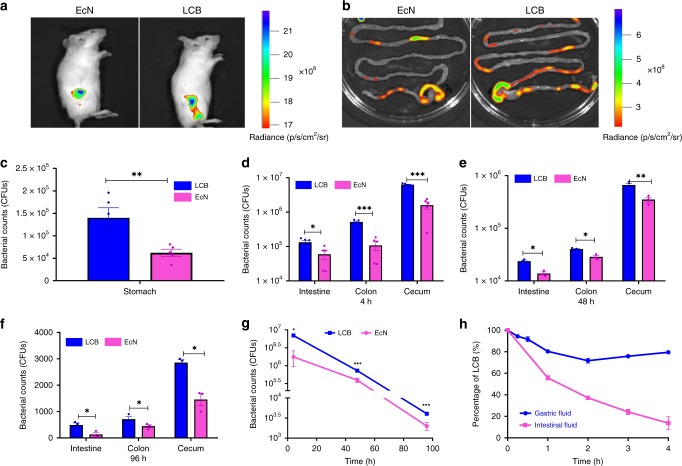
In vivo resistance of LCB against GI tract environment. **a**, **b** Representative IVIS images of mice (**a**) and their intestinal tracts (**b**) after oral gavage of uncoated EcN carrying pBBR1MCS2-Tac-eGFP and LCB for 4 h. **c** Bacterial survival in the stomach at 4 h post-administration. **d**–**f** Bacterial presence in the intestine, colon and cecum at (**d**) 4, (**e**) 48 and (**f**) 96 h post-administration, respectively. **g** Total amount of EcN expressing eGFP retained in the intestinal tract. Each mouse was fed with 1 × 10^8^ CFU of uncoated EcN and LCB by gavage and then sacrificed at 4, 48, and 96 h post-administration. The contents extracted from stomach, intestine, colon and cecum were serially diluted with PBS and then 50 μl of each dilution was spread on solid LB agar plates which were further incubated at 37 °C overnight before counting. **h** The percentage change of LCB over time. LCB were measured by flow cytometry after incubation in either SGF or SIF at 37 °C for different time points. Error bars represent standard deviation (*n* = 5). Significance was assessed using Student’s *t*-test, giving *p* values, *p < 0.05, **p < 0.01, ***p < 0.005.

To determine whether the lipid coating could enhance survival in vivo, colonization of the GI tract was evaluated following oral administration of 10^8^ CFU of coated or uncoated bacteria. The presence of EcN was imaged using an in vivo imaging system (IVIS) at 4 h post-administration. In good agreement with the enhanced in vitro survival of LCB, the coated bacteria displayed increased survival and colonization in the GI tract, particularly the cecum (Fig. 4a, b). To quantify the differences between mice administrated with uncoated EcN and with LCB, the number of EcN in stomach, intestine, colon and cecum were separately evaluated by plate counting. Interestingly, LCB showed almost three-times higher survival in the mouse stomach and more than four-times higher levels in the gut 4 h post-administration when compared with uncoated EcN (Fig. 4c–f). The enhanced reservation in the gut was maintained for up to 4 days post-administration (Fig. 4g). Strikingly, all of the EcN counts in the intestine, colon, and cecum after dosing with LCB were higher than with uncoated bacteria. These data suggest that coating EcN with lipid membranes can greatly improve their oral bioavailability.

We speculated that the improved bioavailability and colonization of LCB could be ascribed to the dynamic behavior of the self-assembled lipid membranes. To verify this hypothesis, we measured the stability of the coating membranes in SGF and SIF. LCB were coated with FITC-labeled lipid membranes and incubated in these fluids for predetermined time periods. As indicated in Fig. 4h and Supplementary Fig. 4a, b, only ~20% of the LCB had disassembled coatings when the incubation time was increased up to 4 h, which exceeds the gastric retention time of mice. This result suggests that the coating membranes are relatively stable in SGF and can be retained during gastric emptying. In the case of SIF, near 90% of the LCB had disassembled coatings following 4 h incubation, which in turn results in exposure of their native cell envelope and recovery of their inherent properties following arrival at intestinal tract. The lowered stability of LCB in SIF could be explained by the rapid bacterial proliferation which promoted the removal of the coating membranes. CLSM images further verify the dynamic character of the coatings (Supplementary Fig. 4c). It’s worth noting that almost no change was found to LCB after 48 h incubation in cold phosphate-buffered saline (PBS) (Supplementary Fig. 4d), suggesting a potential formulation storage condition. Briefly, the coating membranes were stable and remained on the bacteria before division while could be removed rapidly during the division of bacteria. Therefore, the coatings could not affect the growth of the bacteria in media, as which were simply shed off after division. In contrast, the coatings remained intact on the bacteria under environmental assaults because the bacteria stayed dormant and were not able to divide (Supplementary Fig. 4e and f). Consequently, the coatings could protect LCB from gastric fluid during oral delivery, which leaded to significantly increased amount of EcN in vivo. The dynamic self-assembly and disassembly of coating lipid membranes not only improves the oral bioavailability, but also retains the innate bioactivity.

### Treatment of an STm-induced mouse model of colitis

**Fig. 5:**
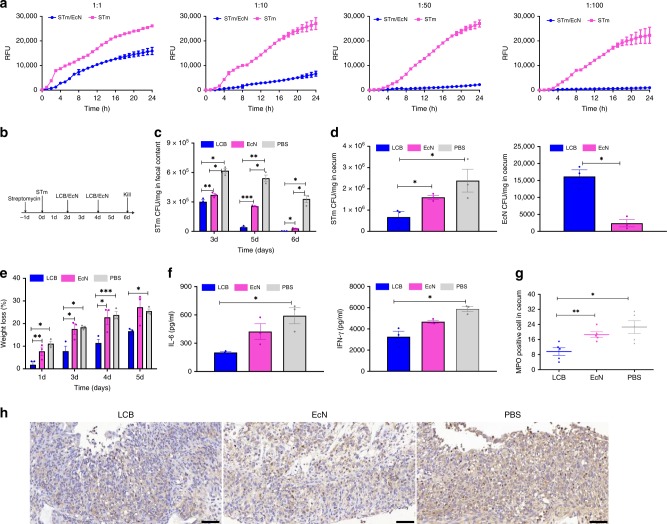
Treatment of a STm-induced mouse model of colitis with LCB. **a** In vitro Bacterial competition between STm and EcN. STm expressing mCherry were co-incubated with EcN in 96 well-plate at various ratios at 37 °C. The relative fluoresce units (RFUs) of mCherry reflecting the growth of STm were recorded by microplate reader at 1 h interval. **b** Experimental design for treatment. Mice were administrated with streptomycin one day before STm infection (1 × 10^9^ CFU) and then orally administrated with either PBS, uncoated EcN, or LCB by gavage at day 2 and 4 post-infection, respectively. Mice were sacrificed at day 6 post-infection. Blinded analysis was performed for ELISA and histopathological analysis. **c** The bacterial counts of STm in feces. At day 3, 5 and 6 post-infection, the feces were collected and resuspended in sterile PBS (0.1 g of fecal material in 2 ml of PBS). Fifty microliters of each sample was spread on selective agar plates and further incubated at 37 °C overnight before counting. **d** The bacterial loads of STm and EcN in the cecum. The cecum was harvested and then homogenized. Fifty microliters of each sample was spread on selective agar plates and further incubated at 37 °C overnight before counting. **e** Weight loss of mice administrated with PBS, uncoated EcN and LCB, respectively. **f** Levels of cytokines including IL-6 and IFN-γ in serum measured by commercially available ELISA kits. **g** Mean MPO positive cells counted in cecum. **h** Typical images of MPO staining of cecum. Error bars represent standard deviation (*n* = 5). Significance was assessed using Student’s *t*-test, giving *p* values, *p < 0.05, **p < 0.01, ***p < 0.005. Scale bar: 50 μm.

Our findings on the in vivo survival of LCB encouraged us to further evaluate whether they could be exploited as enhanced therapeutics for prevention and treatment. Ulcerative colitis, one of the main variants of inflammatory bowel disease, affects about 11.2 million people worldwide and its incidence is increasing^28^. Clinical therapies fail to control symptoms adequately in a large number of patients, adversely affecting quality of life^29,30^. Recently, EcN has been reported to treat STm-induced colitis, as the secreted microcin can inhibit the proliferation of STm^31^. We tested the potency of EcN by co-culturing with STm and found that EcN inhibited the growth of STm in a dose-dependent manner (Fig. 5a and Supplementary Fig. 5a). We next investigated the potential of LCB to treat colitis in an STm-induced mouse model^31^. Mice were administrated with streptomycin one day before STm infection, and then orally administrated with LCB by gavage at day 2 and 4 post-infection (Fig. 5b). Both uncoated EcN and PBS were separately dosed as controls. The excreted STm in feces were enumerated at the indicated time points. As shown in Fig. 5c, the amounts of STm reduced dramatically and continuously after treating with LCB, with a 10 to 100-times reduction compared to that of mice treated with uncoated EcN and PBS at day 4 post-treatment. Comparing to the controls, treatment with LCB resulted in 2 to 4-times less STm in the cecum, which could be explained by the colonization of a large number of EcN (Fig. 5d). The enhanced inhibition of STm after treating with LCB also resulted in less weight loss (Fig. 5e). In addition, blinded enzyme-linked immunosorbent assay (ELISA) and myeloperoxidase (MPO) staining were performed to assess the inflammation of mice infected by STm. In contrast to those treated with uncoated EcN and PBS, treating mice with LCB significantly reduced inflammation as reflected by the lower levels of cytokines in serum, including interleukin-6 (IL-6) and tumor necrosis factor-γ (IFN-γ) (Fig. 5f), as well as less MPO positive cells in cecal lesions (Fig. 5g, h).

**Fig. 6:**
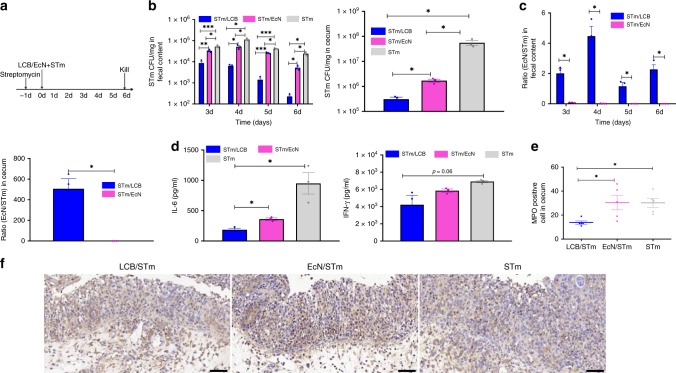
Prevention of a STm-induced mouse model of colitis with LCB. **a** Experimental design for prevention. Mice were co-administrated with an equal amount (1 × 10^9^ CFU) of STm and LCB. Equivalent doses of uncoated EcN and blank PBS were administrated as controls. Mice were sacrificed at day 6 post-administration. **b** The bacterial counts of STm in feces and cecum. **c** The ratios of EcN/STm in feces and cecum. **d** Levels of cytokines including IL-6 and IFN-γ in serum. **e** Mean MPO positive cells counted in cecum. **f** Typical images of MPO staining of cecum. Error bars represent standard deviation (*n* = 5). Significance was assessed using Student’s *t*-test, giving *p* values, *p < 0.05, **p < 0.01, ***p < 0.005. Scale bar: 50 μm.

In view of the increased efficacy to treat colitis, we next turned our attention to explore the potential of LCB for prevention of disease by evaluating the in vivo competition between these two strains. Mice were co-administrated with an equal amount of STm and LCB (Fig. 6a). Equivalent doses of uncoated EcN and blank PBS were administrated as controls. In comparison with the controls, mice co-administrated with LCB exhibited dramatically reduced survival and colonization of STm (Fig. 6b), which was ascribed to the increased presence of EcN (Supplementary Fig. 5b and c). In particular, the ratio of EcN/STm in feces excreted from mice co-administrated with LCB was 25 to 8000-times higher than those of mice treated with uncoated EcN (Fig. 6c). Moreover, mice co-treated with LCB showed up to 3100-fold higher ratios of EcN/STm in the cecum. Consequently, the superior suppression achieved by dosing with LCB alleviated inflammatory symptoms, as indicated by significantly reduced weight loss (Supplementary Fig. 5d), lower levels of IL-6 and IFN-γ in serum (Fig. 6d), and less MPO positive cells in lesions (Fig. 6e, f).

### Treatment of a DSS-induced mouse model of colitis

**Fig. 7:**
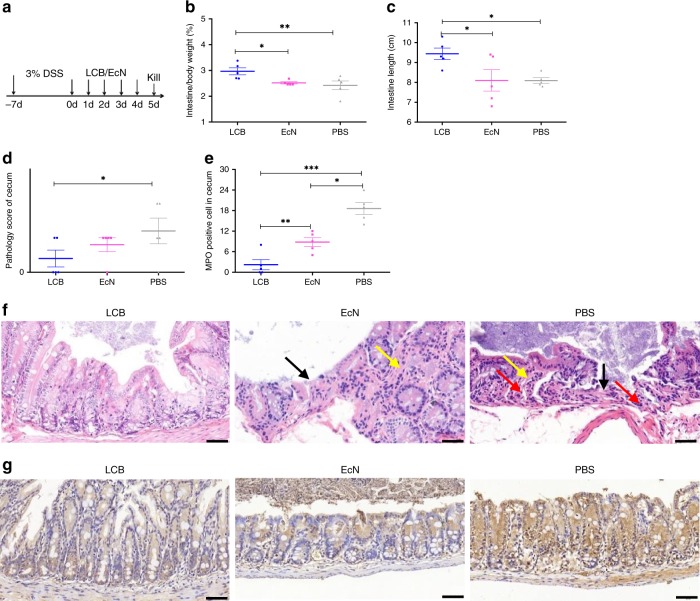
Treatment of a DSS-induced mouse model of colitis with LCB. **a** Experimental design for treatment. Mice were fed with 3% DSS for 7 days to develop colitis, and then administrated with 1 × 10^8^ CFU of LCB daily for a further 5 days. Uncoated EcN and PBS were used as controls. The intestinal tract of treated mice was harvested for blinded histopathological analysis 5 days post-treatment. **b**, **c** The (**b**) weight and (**c**) length of the intestine tract 5 days post-treatment. **d** Histopathology scores of ceca after treatment with PBS, uncoated EcN and LCB, respectively. **e**, **f** Representative images of (**e**) H&E and (**f**) MPO staining of ceca. **g** Mean MPO positive cells counted in ceca. Black arrow: shedding of mucosal epithelium cells; Yellow arrow: disappearance and replacement of intestinal glands in lamina propria; Red: Lymphocyte infiltration. Error bars represent standard deviation (*n* = 5). Significance was assessed using Student’s *t*-test, giving *p* values, *p < 0.05, **p < 0.01, ***p < 0.005. Scale bar: 50 μm.

Having confirmed the enhanced prevention and treatment of LCB in STm-induced colitis, we further examined the potency of LCB in a DSS-induced colitis model^22,31^. Mice were fed with 3% DSS for 7 days to develop colitis and then administrated with LCB daily for another 5 days (Fig. 7a). Uncoated EcN and PBS were used as controls. The intestinal tract of treated mice was harvested for blinded histopathological analysis 5 days post-treatment. Compared to mice dosed with LCB, direct reductions in intestinal weight and length were observed from mice treated with uncoated EcN and PBS (Fig. 7b and c). The disease severity of ceca from mice treated with LCB diminished significantly (mean score 0.4) in comparison to mice dosed with EcN (mean score 0.8) and PBS (mean score 1.2) (Fig. 7d). Representative images of haematoxylin and eosin (H&E) staining in Fig. 7f show that no histological damage, such as shedding of mucosal epithelium cells and disappearance of intestinal glands in lamina propria, could be observed in the cecum from mice treated with LCB. Additionally, the average number of MPO positive cells was also significantly lower than those in EcN and PBS groups (Fig. 7e, g).

**Fig. 8:**
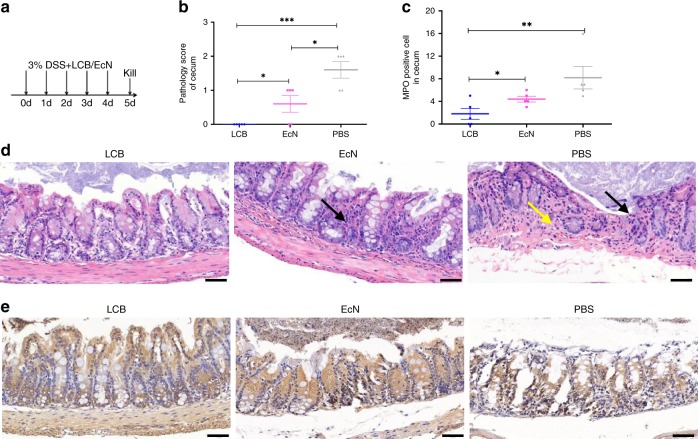
Prevention of a DSS-induced mouse model of colitis with LCB. **a** Experimental design for prevention. Mice were fed with 3% DSS and LCB together for 5 days. Uncoated EcN and PBS were used as controls. Mice were sacrificed for analysis at day 5 post-administration. **b** Histopathology scores of ceca after treatment with PBS, uncoated EcN and LCB, respectively. **c** Mean MPO positive cells counted in ceca. **d**, **e** Representative images of (**d**) H&E and (**e**) MPO of ceca. Black arrow: shedding of mucosal epithelium cells; Yellow arrow: disappearance and replacement of intestinal glands in lamina propria. Error bars represent standard deviation (*n* = 5). Significance was assessed using Student’s *t*-test, giving *p* values, *p < 0.05, **p < 0.01, ***p < 0.005. Scale bar: 50 μm.

Finally, we performed prevention trials according to therapeutic design displayed in Fig. 8a. Mice were fed with 3% DSS and LCB together for 5 days. Controls using uncoated EcN and PBS were also carried out. As shown in Fig. 8b, co-treatment with LCB showed significant impact on the reduction of disease severity in the cecum (mean score 0). Meanwhile, histological inflammation was only found in mice treated with uncoated EcN and PBS (Fig. 8d). Furthermore, a significant decrease of MPO positive cells in lesions was observed from mice co-treated with LCB (Fig. 8c, e).

In summary, we have described a simple yet highly efficient method to generate super gut microbes by biointerfacial supramolecular self-assembly. The preparation can be performed in less than 15 min by simply vortexing with FDA approved lipids. The resultant bacteria exhibit: significantly improved survival against various extreme conditions; almost unchanged viability and bioactivity; and enhanced oral efficacies in prevention and treatment. We anticipate the use of this method to generate a myriad of super gut microbes for preventing and treating various diseases.

## Methods

### Materials and strains

*Escherichia coli* Nissle 1917, *Bacillus Subtillus*, *Enterococcus faecalis*, *Staphylococcus aureus*, and *Salmonella typhimurium* were purchased from China general microbiological culture collection center (GMCC, China). Plasmids pBBR1MCS2-Tac-mCherry (Kanamycin resistant), pBBR1MCS2-Tac-eGFP (Kanamycin resistant), and pMD18-luxCDABE (Ampicillin resistant), and all other reagents were purchased from domestic suppliers and used as received. All the bacteria were grown in LB medium at 37 °C with suitable antibiotics.

### Preparation of LCB

LCB were prepared according to the reported method with minor modification^32^. Briefly, 1.5 ml of bacterial sub-culture were washed and resuspended in 1 ml of ice cold calcium phosphate solution containing 12.5 mM of CaCl_2_. DOPA (Avanti Polar Lipids, Alabaster, AL, USA) and cholesterol (Sinopharm, Beijing, China) were dissolved in 2 ml of chloroform at a 4:1 molar ratio. The resultant solution was dried at room temperature using a rotary evaporator, resulting in a lipid film. The obtained film was hydrated in 1 ml of bacterial solution and vortexed for 15 min and then stored at 4 °C for further characterization. FITC-labeled coating was prepared by co-assembly with FITC-mPEG2000-DSPE (10% molar ratio to DOPA). Nile Red-labeled coating was prepared by pre-dissolving Nile Red in the chloroform solution.

### Characterization of LCB

The morphology of LCB was visualized using a transmission electron microscopy (HITACH, Japan). A drop of LCB solution was deposited onto a carbon-coated copper grid. The sample was then washed with ddH_2_O twice, each of which lasted for 5 min. Subsequently, the sample was dried completely in air before observation. The average size and apparent zeta potential of LCB were determined by dynamic light scattering (Malvern Zetasizer nano ZS, UK) measurements. The lipid coating labeled with FITC was examined by using flow cytometry (Beckman CytoFlex, USA). Additionally, the coating and decoating of eGFP-expressed EcN was observed by laser scanning confocal microscopy (Leica TCS SP8, German).

### Growth curves of LCB

Bacteria were collected and washed with ice cold PBS before mixing with lipid membranes as described in the section of preparation of LCB. Both uncoated EcN and LCB were diluted to reach an optical density (OD) value of ~0.15 in LB, and incubated at 37 °C with gently shaking. The OD value of cultures was recorded at 600 nm at 0.5 h intervals by microplate reader (BioTek, USA).

### Cell viability assay

Cell viability was examined by a CCK-8 (Beyotime, China). Both uncoated EcN and LCB were diluted to an OD_600_ value of ~0.5 in LB medium, LB supplemented with 50% serum, or PBS. 190 μl of each culture medium was inoculated into a 96-well plate and cultured at 37 °C without shaking. Ten microlitres of CCK-8 solution was added into each well. The OD value of cultures was recorded at 450 nm at 1 h intervals. A multi-detection microplate reader (BioTek, USA) was used to measure the absorbance at 450 nm.

### Resistance assay

Equal amount of LCB and uncoated EcN carrying pBBR1MCS2-Tac-mCherry were resuspended into 200 μl medium supplemented with either antibiotic combination of ampicillin and apramycin (7.5 mg ml^−1^), strong acid solution (0.85% NaCl, pH 2.0), strong alkali solution (0.85% NaCl, pH 11.0), 30% ethanol (v/v), 50% ethanol (v/v), bile salts (0.3 mg ml^−1^), SGF supplemented with pepsin (pH 1.2), or SIF containing trypsin (pH 6.8) and incubated at 37 °C with gently shaking. At predetermined time points, 50 μl of each sample was taken, washed with fresh LB medium, and spread on LB agar plates containing 50 μg ml^−1^ kanamycin. The colonies were counted after 24 h incubation at 37 °C. Samples were withdrawn at predetermined time points and examined by TEM.

### Stability of LCB in simulated GI fluids

Hundred microliters of LCB labeled with FITC were resuspended into 900 μl of SGF containing 10 g L^−1^ of pepsin in 0.85% NaCl solution (HCl, pH 1.2) or SIF containing 10 g L^−1^ of trypsin in KH_2_PO_4_ solution (NaOH, pH 6.8), and incubated at 37 °C. Hundred microliters of each sample was taken at predetermined time points and examined by flow cytometry. FITC-labeled LCB cultured in 4 °C PBS was also performed to evaluate the stability of lipid coatings on the bacteria. All the solutions were sterilized by 0.22 μm filter.

### GI retention of LCB

All the animal procedures are complied with the guidelines of the Shanghai Medical Experimental Animal Care. Animal protocols were approved by the Institutional Animal Care and Use Committee of Shanghai Jiao Tong University School of Medicine. The experiments were performed on 6–8 weeks-old female ICR mice. Three mice for each time points per group were used. To evaluate the bacterial retention of LCB in the GI tract, 1 × 10^8^ CFU of coated bacteria were administered intragastrically. The mice were sacrificed at 4, 48, and 96 h post-administration. Uncoated EcN were used as a control. The contents in the GI tract, including stomach, intestine, colon and cecum, were serially diluted with PBS and 50 μl of each dilution was spread on solid LB agar plates, which were further incubated at 37 °C overnight before counting. The survivals in GI tract of mice were also imaged by IVIS system at 4 h post oral gavage of LCB or uncoated EcN.

### In vitro bacterial competition

EcN (Ampicillin resistant) and STm (Kanamycin resistance, expressing mCherry) were separately grown at 37 °C for 12 h and subsequently diluted to an OD value of 1.0. STm and EcN were then mixed in a serial of ratios (1:1, 1:10, 1:50, 1:100) and incubated at 37 °C with shaking. Fifty microliters of each sample was diluted and spread into selective LB agar plates containing 50 μg ml^−1^ of kanamycin at determined time points and incubated at 37 °C for 24 h before bacterial counting. The competition between STm and EcN was also measured continuously for 24 h by microplate reader. STm and EcN were mixed with a serial of ratios (1:1, 1:10, 1:50, 1:100) and 150 μl of each mixture were incubated at 37 °C with continuously shaking. The relative fluoresce units (RFUs) of mCherry expressed by STm were recorded at 580/610 nm at 1 h interval.

### In vivo bacterial competition

Female C57BL/6 mice at 5–6 weeks of age were treated with 100 μl of streptomycin solution (200 mg ml^−1^) before infection. The following day mice were grouped randomly and treated with wither 1 × 10^9^ CFU of STm, STm together with uncoated EcN (1:1 ratio of 1 × 10^9^ CFU), or 1 × 10^9^ CFU of STm and LCB. At day 3, 4, and 5 post-infection, the fecal were collected and resuspended into sterile PBS. Fifty microliters of each sample was spread on selective agar plates and further incubated at 37 °C overnight before counting.

### Dextra sulfate sodium (DSS)-induced mouse model of colitis

ICR mice at 5–6 weeks of age were treated with 3% DSS salt (reagent grade; MW 36,000–50,000 kDa; Sangon, China) in sterile drinking water for 7 days as described previously^33,34^. Then the mice were randomly assigned to different treatment groups and administered with LCB or EcN (1 × 10^8^ CFU/mouse/day) or PBS by gavage for 5 days^22^. The colon and cecum were harvested and fixed in 4% formalin for blinded histopathology analysis.

### Salmonella typhimurium-induced mouse model of colitis

The *Salmonella typhimurium*-induced colitis was established as described previously^31^. Female C57BL/6 mice at 5–6 weeks of age were treated with 100 μl of streptomycin solution (200 mg ml^−1^) prior to infection by orally inoculating with 1 × 10^9^ CFU of *Salmonella*. At day 2 and 4 post-infection, mice were treated with 1 × 10^9^ CFU of LCB or EcN. PBS was used as a control. Mice were sacrificed at day 6 post-infection and tissue samples were harvested and fixed in 4% formalin for blinded histopathology analysis.

### Cytokine assay

The treated mice were sacrificed at day 6 post-infection. one microliter of blood from each mouse was withdrawn through eye socket and stored in a 1.5 ml Eppendorf tube without EDTA and incubated at 37 °C for 0.5 h. The serum was isolated by centrifugation at 10,000 × *g* for 5 min and then assayed for IL-6 and IFN-γ using commercially available ELISA kits (MultiSciences Biotech, China).

### Histopathology analysis

Tissue samples were fixed in 4% formalin, processed according to standard procedures for paraffin embedding, sectioned at 4 μm, followed by hematoxylin and eosin (H&E) staining and MPO staining. The tissues were scanned by 3D HISTECH Pannoramic 250 (3DHISTECH, Hungary). The pathology score of cecal samples was determined by blinded examination of cecal sections from a board-certified pathologist using previously published methods^30,35^. Each section was evaluated for the presence of mono-nuclear cell infiltration, polymorphonuclear cell infiltration, epithelial hyperplasia, and epithelial injury. The scores are independently graded as absent (0), mild (1), moderate (2), severe (3), giving a total score of 0 to 12. MPO activities were measured by MPO staining. The MPO positive cells in brown were counted by blinded examination of cecal sections from a board-certified pathologist.

### Online content

Methods, along with any additional Extended Data display items and Source Data, are available in the online version of the paper; references unique to these sections appear only in the online paper.

### Reporting summary

Further information on research design is available in the Nature Research Reporting Summary linked to this article.

## Supplementary information

Supplementary Information

Reporting Summary

## Data Availability

All data presented in the paper are available from the authors upon reasonable request.

## References

[1] Sekirov I, Russell SL, Antunes LC, Finlay BB (2010). Gut microbiota in health and disease. Physiol. Rev..

[2] Brussow H (2016). Biome engineering-2020. Microb. Biotechnol..

[3] Valdes AM, Walter J, Segal E, Spector TD (2018). Role of the gut microbiota in nutrition and health. BMJ.

[4] Alexander JL (2017). Gut microbiota modulation of chemotherapy efficacy and toxicity. Nat. Rev. Gastroenterol. Hepatol..

[5] Eom T, Kim YS, Choi CH, Sadowsky MJ, Unno T (2018). Current understanding of microbiota- and dietary-therapies for treating inflammatory bowel disease. J. Microbiol.

[6] Knip M, Siljander H (2016). The role of the intestinal microbiota in type 1 diabetes mellitus. Nat. Rev. Endocrinol..

[7] Lau LHS, Wong SH (2018). Microbiota, Obesity and NAFLD. Adv. Exp. Med. Biol..

[8] Riaz QU, Masud T (2013). Recent trends and applications of encapsulating materials for probiotic stability. Crit. Rev. Food Sci. Nutr..

[9] Jandhyala SM (2015). Role of the normal gut microbiota. World J. Gastroenterol..

[10] Jiang T (2017). Oral delivery of probiotics in poultry using pH-sensitive tablets. J. Microbiol. Biotechnol..

[11] Li Z (2018). Biofilm-inspired encapsulation of probiotics for the treatment of complex infections. Adv. Mater..

[12] Kwiecien, I. & Kwiecien, M. Application of polysaccharide-based hydrogels as probiotic delivery systems. *Gels* **4**, 47 (2018). 10.3390/gels4020047 PMC6209284 30674823

[13] Anselmo AC, McHugh KJ, Webster J, Langer R, Jaklenec A (2016). Layer-by-layer encapsulation of probiotics for delivery to the microbiome. Adv. Mater..

[14] McKay R (2018). A platform of genetically engineered bacteria as vehicles for localized delivery of therapeutics: toward applications for Crohn’s disease. Bioeng. Transl. Med..

[15] Kurtz Caroline B., Millet Yves A., Puurunen Marja K., Perreault Mylène, Charbonneau Mark R., Isabella Vincent M., Kotula Jonathan W., Antipov Eugene, Dagon Yossi, Denney William S., Wagner David A., West Kip A., Degar Andrew J., Brennan Aoife M., Miller Paul F. (2019). An engineered E. coli Nissle improves hyperammonemia and survival in mice and shows dose-dependent exposure in healthy humans. Science Translational Medicine.

[16] Palmer JD (2018). Engineered probiotic for the inhibition of salmonella via tetrathionate-induced production of microcin H47. ACS Infect. Dis..

[17] Hwang IY (2017). Engineered probiotic *Escherichia coli* can eliminate and prevent *Pseudomonas aeruginosa* gut infection in animal models. Nat. Commun..

[18] Solanki HK (2013). Development of microencapsulation delivery system for long-term preservation of probiotics as biotherapeutics agent. BioMed. Res. Int..

[19] Timmis K, Timmis JK, Brussow H, Fernandez LA (2019). Synthetic consortia of nanobody-coupled and formatted bacteria for prophylaxis and therapy interventions targeting microbiome dysbiosis-associated diseases and co-morbidities. Microb. Biotechnol..

[20] Jacobi CA, Malfertheiner P (2011). *Escherichia coli* Nissle 1917 (Mutaflor): new insights into an old probiotic bacterium. Dig. Dis..

[21] Isabella VM (2018). Development of a synthetic live bacterial therapeutic for the human metabolic disease phenylketonuria. Nat. Biotechnol..

[22] Kamada N (2005). Nonpathogenic *Escherichia coli* strain Nissle1917 prevents murine acute and chronic colitis. Inflamm. Bowel Dis..

[23] Schultz M (2008). Clinical use of *E. coli* Nissle 1917 in inflammatory bowel disease. Inflamm. Bowel Dis..

[24] Sonnenborn, U. & Schulze, J. The non-pathogenic *Escherichia coli* strain Nissle 1917 – features of a versatile probiotic. *Microb. Ecol. Health Dis.* **21**, 122–158 (2009).

[25] Cao Z, Cheng S, Wang X, Pang Y, Liu J (2019). Camouflaging bacteria by wrapping with cell membranes. Nat. Commun..

[26] Dharan S, Hugonnet S, Sax H, Pittet D (2003). Comparison of waterless hand antisepsis agents at short application times: raising the flag of concern. Infect. Control Hosp. Epidemiol..

[27] Morton HE (1950). The relationship of concentration and germicidal efficiency of ethyl alcohol. Ann. N. Y. Acad. Sci..

[28] Ungaro R, Mehandru S, Allen PB, Peyrin-Biroulet L, Colombel JF (2017). Ulcerative colitis. Lancet.

[29] Hoivik ML (2012). Health-related quality of life in patients with ulcerative colitis after a 10-year disease course: results from the IBSEN study. Inflamm. Bowel Dis..

[30] Zhang S (2015). An inflammation-targeting hydrogel for local drug delivery in inflammatory bowel disease. Sci. Transl. Med..

[31] Sassone-Corsi M (2016). Microcins mediate competition among Enterobacteriaceae in the inflamed gut. Nature.

[32] Chen J (2016). Oncolytic adenovirus complexes coated with lipids and calcium phosphate for cancer gene therapy. ACS Nano.

[33] Bain CC (2013). Resident and pro-inflammatory macrophages in the colon represent alternative context-dependent fates of the same Ly6Chi monocyte precursors. Mucosal Immunol..

[34] Liu WS, Chen MC, Chiu KH, Wen ZH, Lee CH (2012). Amelioration of dextran sodium sulfate-induced colitis in mice by Rhodobacter sphaeroides extract. Molecules.

[35] Ermann J, Staton T, Glickman JN, de Waal Malefyt R, Glimcher LH (2014). Nod/Ripk2 signaling in dendritic cells activates IL-17A-secreting innate lymphoid cells and drives colitis in T-bet-/-.Rag2-/- (TRUC) mice. Proc. Natl Acad. Sci. USA.

