# Supplementary material for: Biointerfacial self-assembly generates lipid membrane coated bacteria for enhanced oral delivery and treatment
Source: Nat Commun. 2019 Dec 19;10:5783. doi: 10.1038/s41467-019-13727-9 (PMC6923387; doi:10.1038/s41467-019-13727-9)
Supplement: Supplementary file 1 — Supplementary Information [file 41467_2019_13727_MOESM1_ESM.pdf]

Supplementary Information

Biointerfacial self-assembly generates lipid membrane coated bacteria for enhanced oral delivery and treatment

Cao et al.

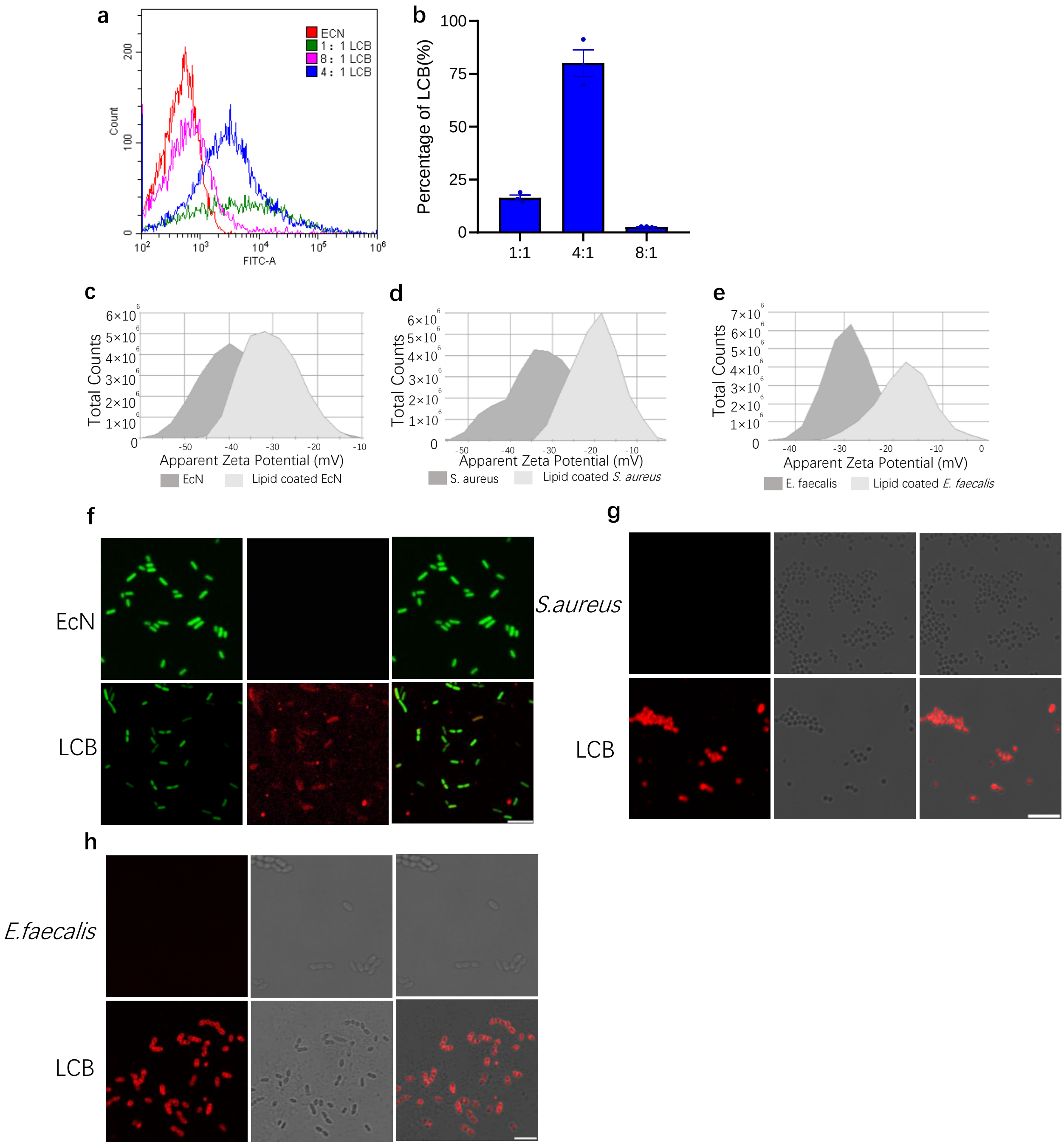

Supplementary Figure 1 Characterization of LCB. a, b, The optimizations of molar ratio (DOPA to cholesterol) on lipid composition measured by flow cytometry. Error bars represent standard deviation (n=3). Significance was assessed using Student's t-test, giving p values, $p<0.05$, *, $p<0.01$, **, $p<0.005$, ***. **c-e**, Apparent zeta potential of LCB and uncoated bacteria measured by DLS. **f**, Representative LSCM images of EcN coated with lipid membranes. The red channel shows lipid membranes labelled with Nile Red, the green channel shows EcN expressing eGFP and the merge (light orange) shows LCB. Scale bar, 5 µm. **g**, Typical LSCM images of *S.aureus* coated with lipid membranes. The red channel shows lipid membranes labelled with Nile Red, Scale bar, 10 µm. **h**, Representative LSCM images of *E.faecalis* coated with lipid membranes. The red channel shows lipid membranes labelled with Nile Red, Scale bar, 5 µm.

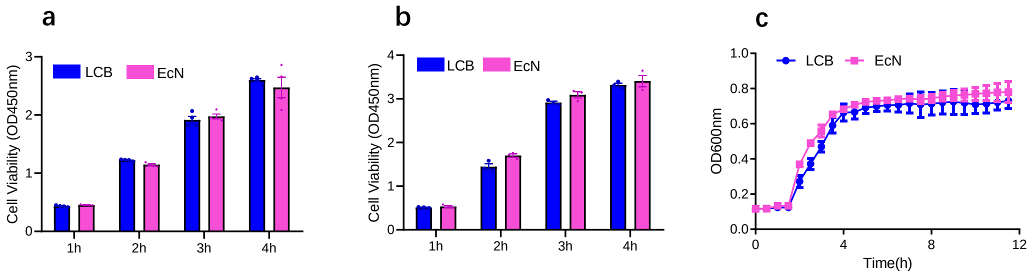

Supplementary Figure 2 Bacterial viability analysis. a, **b**, Bacteria were incubated in (**a**) LB and (**b**) LB with 50% serum at 37 °C and bacterial viability was monitored by measuring OD450 at 1 hour interval. Error bars represent standard deviation (n=3). **c**, Growth curves of uncoated EcN and LCB cultured in LB with 50% serum at 37 °C and the OD_{600} was recorded at 30 min intervals using a microplate reader.

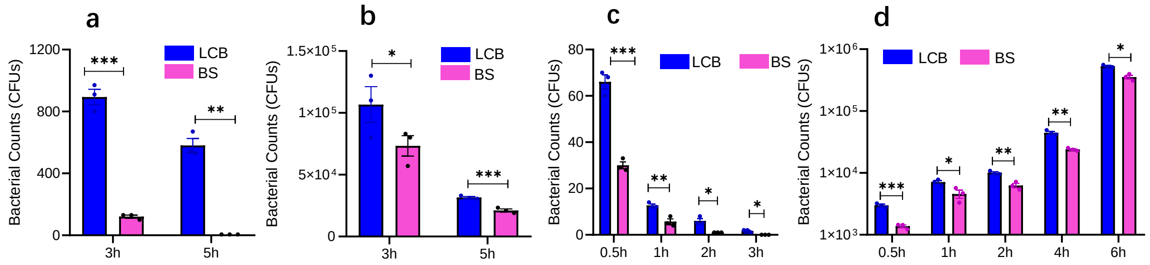

Supplementary Figure 3 ***In vitro*** **resistance of coated BS against environmental assaults. a-d**, Equal amounts of BS and coated BS were exposed to the following: (**a**) an antibiotic cocktail of ampicillin and apramycin, (**b**) bile salts (0.3 mg ml^{-1}), (**c**) SGF, and (**d**) SIF at 37 ℃. After the indicated time points, 50 µl of each sample was washed twice with fresh LB, spread onto LB agar plates and incubated at 37 ℃ for 24 hours before bacterial counting. Error bars represent standard deviation (n=3). Significance was assessed using Student's t-test, giving p values, $p<0.05$, *; $p<0.01$, **; $p<0.005$, ***.

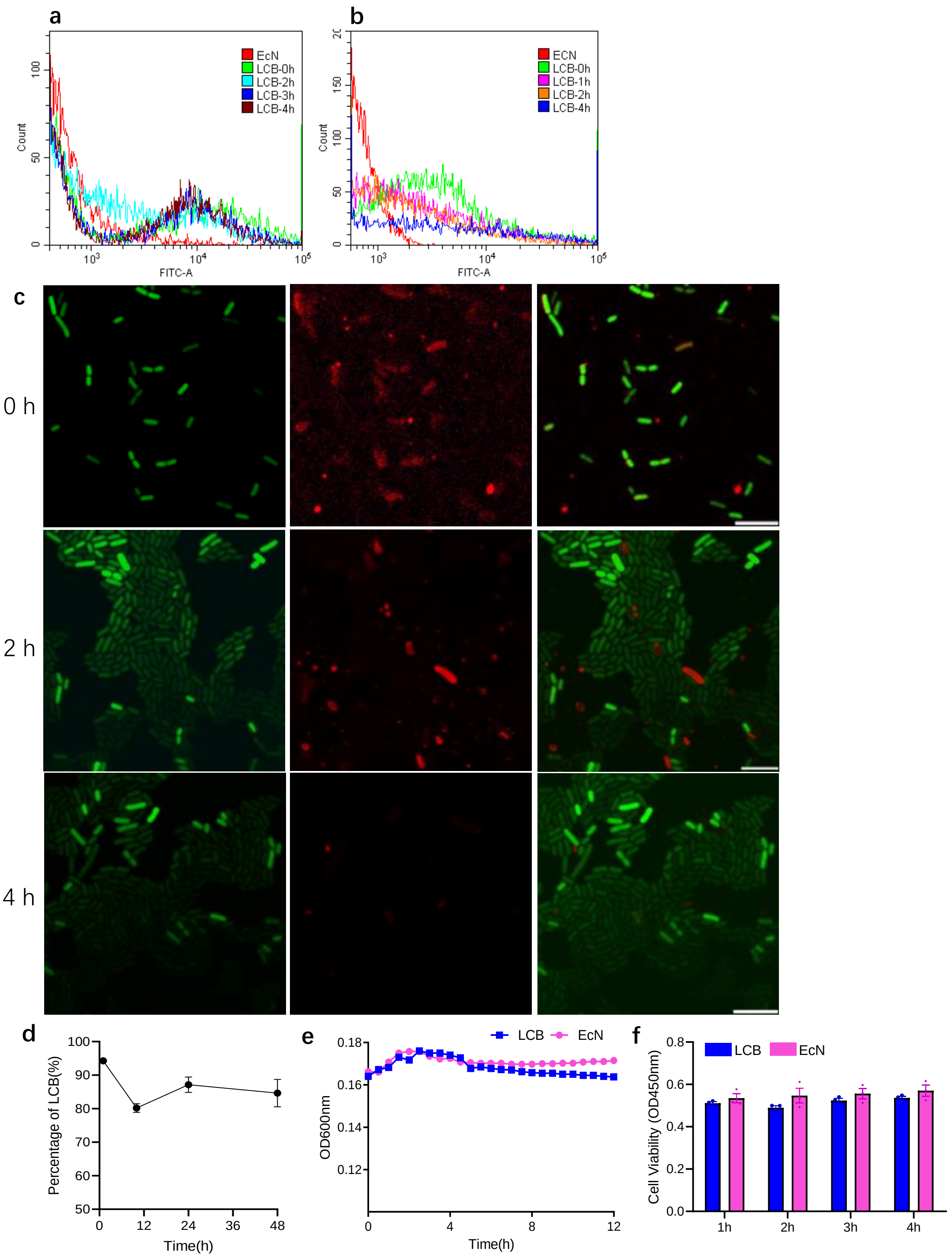

Supplementary Figure 4 Stability of the lipid coating membranes. a, **b**, Flow cytometric analysis of FITC-labelled LCB after incubation in (**a**) SGF and (**b**) SIF. **c**, Representative LSCM images of LCB after culturing in LB agar pad at 37 °C for indicated time points. The red channel shows the coating membranes labelled with Nile Red and the green channel shows EcN expressing eGFP. Scale bar, 5 μm. **d**, Stability of LCB in ice cold PBS for predetermined time points. Error bars represent standard deviation (n=3). **e**, Growth curves of uncoated EcN and LCB cultured in PBS at 37 °C and the OD_{600} was recorded at 30 min intervals using a microplate reader. **f**, Bacteria were incubated in PBS at 37 °C and bacterial viability was monitored by measuring OD_{450} at 1 hour interval.

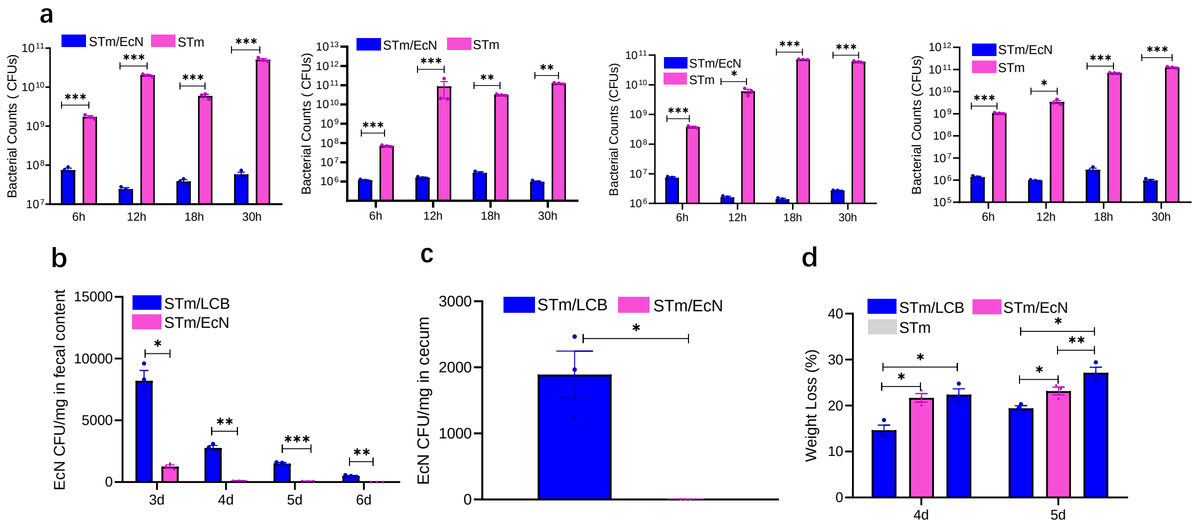

Supplementary Figure 5 Bacterial competition. a, STm were co-incubated with EcN in LB at various ratios at 37 ℃. The survivals of STm were examined at indicated time points by bacterial counting on selective agar. **b**, The bacterial counts of EcN in feces. **c**, The ratios of EcN/STm in feces. Mice were orally administered with a 1:1 ratio (1 × 10^9 CFU) of STm and LCB. Equal amount of STm and uncoated EcN was used as a control. At day 3, 4, 5 and 6 post-infection, the feces were collected and resuspended into sterile PBS (0.1g of fecal material into 2 ml PBS). The bacterial loads of EcN were determined by spreading 50 μl of each dilution on selective agar plates. **d**, The weight loss of mice at day 4 and 5 post-infection. Error bars represent standard deviation (n=3). Significance was assessed using Student's t-test, giving p values, $p<0.05$, *, $p<0.01$, **, $p<0.005$, ***.
